# Suppression of choriocarcinoma invasion and metastasis following blockade of BDNF/TrkB signaling

**DOI:** 10.1002/cam4.158

**Published:** 2013-11-07

**Authors:** Kazuhiro Kawamura, Nanami Kawamura, Naoki Okamoto, Motomu Manabe

**Affiliations:** 1 Department of Obstetrics and Gynecology, St. Marianna University School of MedicineKanagawa, 216-8511, Japan; 2 Department of Obstetrics and Gynecology, Akita University Graduate School of MedicineAkita, 010-8543, Japan; 3 Department of Dermatology and Plastic Surgery, Akita University Graduate School of MedicineAkita, 010-8543, Japan

**Keywords:** BDNF, choriocarcinoma, invasion, metastasis, TrkB

## Abstract

Brain-derived neurotrophic factor (BDNF) acts through its cognate receptor tyrosine kinase-B (TrkB) to regulate diverse physiological functions in reproductive and other tissues. In normal and malignant trophoblastic cells, the BDNF/TrkB signaling promotes cell growth. Due to the highly malignant nature of choriocarcinoma, we investigated possible involvement of this system in choriocarcinoma cell invasion and metastasis. We demonstrated that treatment of cultured choriocarcinoma cells, known to express both BDNF and TrkB, with a soluble TrkB ectodomain or a Trk receptor inhibitor K252a suppressed cell invasion accompanied with decreased expression of matrix metalloproteinase-2, a cell invasion marker. In vivo studies using a tumor xenograft model in athymic nude mice further showed inhibition of cell invasion from tumors to surrounding tissues following the suppression of endogenous TrkB signaling. For an in vivo model of choriocarcinoma metastasis, we performed intravenous injections of JAR cells expressing firefly luciferase into severe combined immunodeficiency (SCID) mice. Treatment with K252a inhibited metastasis of tumors to distant organs. In vivo K252a treatment also suppressed metastatic tumor growth as reflected by decreased cell proliferation and increased apoptosis and caspases-3/7 activities, together with reduced tissue levels of a tumor marker, human chorionic gonadotropin-β. In vivo suppression of TrkB signaling also led to decreased expression of angiogenic markers in metastatic tumor, including cluster of differentiation 31 and vascular endothelial growth factor A. Our findings suggested essential autocrine/paracrine roles of the BDNF/TrkB signaling system in choriocarcinoma invasion and metastasis. Inhibition of this signaling could serve as the basis to develop a novel therapy for patients with choriocarcinoma.

## Introduction

Brain-derived neurotrophic factor (BDNF) belongs to the neurotrophin family known to activate the high-affinity tyrosine kinase-B (TrkB) receptor [1]. Following BDNF binding, TrkB activates multiple signaling pathways, including phosphatidylinositol 3-kinase, mitogen-activated protein kinase/extracellular signal-regulated kinase (MAPK/ERK), phospholipase C-γ, and protein kinase C to regulate cell proliferation, differentiation, and survival in different cell types [1, 2]. Although neurotrophins were originally discovered in the central nervous system and are important for its development [3], they also play physiological roles in nonneuronal tissues [1–4]. In reproductive tissues, we detected the expression of TrkB and its ligands, BDNF and neurotrophin-4/5, in trophectoderm cells of blastocyst-stage embryos. We also demonstrated the stimulatory effects of TrkB signaling on the proliferation and survival of trophectoderm cells before implantation in mice [5]. After implantation, TrkB and its ligands continue to be expressed in different placental trophoblasts, and the TrkB signaling promoted trophoblast growth and survival during murine placental development in an autocrine/paracrine manner [6]. In human, we demonstrated potential autocrine/paracrine roles of the BDNF/TrkB signaling system for trophoblast growth during both ectopic and intrauterine normal pregnancies [7]. This system is also important for the development of hydatidiform mole, a gestational trophoblastic disease [8].

Accumulating evidence indicates that altered neurotrophin signaling through TrkB promotes malignant tumor formation and metastasis [9]. TrkB, sometimes in conjunction with its ligand, BDNF, is often overexpressed in different human cancers including neuroblastomas, Wilms's tumor, and prostate and pancreatic adenocarcinomas, as well as multiple myeloma, a nonsolid tumor [9]. Overexpression of BDNF/TrkB was also found to associate with increased metastatic potential of several malignant tumors [9]. Treatment with BDNF promoted cell invasion of diverse cancer cells [10–14], whereas suppression of BDNF/TrkB signaling abrogated invasion of hepatocellular carcinoma cells [15], migration and invasion of head and neck squamous cell carcinoma [13], as well as transitional cell carcinoma [16]. Furthermore, TrkB signaling was shown to be a potent stimulator of angiogenesis [17], and this signaling mediates anoikis resistance, which is important for tumor metastasis [18, 19].

Choriocarcinoma is a highly anaplastic, invasive, and metastatic tumor derived from trophoblast cells. Despite well-established first-line chemotherapy, 5–25% of choriocarcinoma patients showed incomplete responses, or relapsed due to tumor remission. In our earlier studies, we demonstrated important autocrine roles of the BDNF/TrkB signaling system in choriocarcinoma cell growth [20]. Inhibition of endogenous TrkB signaling in choriocarcinoma cells resulted in the suppression of cell proliferation and survival [20]. However, the roles of TrkB signaling system in choriocarcinoma invasion and metastasis remain to be determined. Here, we extended our studies and showed the autocrine roles of the TrkB signaling system in choriocarcinoma cell invasion in vitro and in vivo using soluble ectodomains of TrkB and small molecular weight Trk receptor inhibitors. In an in vivo animal model of choriocarcinoma metastasis, we further demonstrated the anticancer potential of the Trk receptor inhibitors in suppressing choriocarcinoma angiogenesis, metastasis, and metastatic tumor growth in vivo.

## Material and Methods

### Cells

The choriocarcinoma cell lines, JAR and BeWo, were purchased from American Type Culture Collection (Manassas, VA) with authentication by short tandem repeat profiling and maintained in Dulbecco's modified Eagle medium (DMEM)/F12 (Invitrogen, Carlsbad, CA) supplemented with 10% fetal bovine serum (FBS) (Invitrogen), penicillin (100 U/mL), and streptomycin (100 μg/mL) at 37°C in a 5% CO_2_ atmosphere. Cells with <10 passages numbers were used for all experiments. Among different choriocarcinoma cell lines, JAR cells were used in this study due to its high production of the tumor marker, human chorionic gonadotropin (hCG)-β [20].

To establish JAR cell lines expressing firefly luciferase, cells were transfected with the firefly luciferase vector (pGL4.50; Promega KK, Tokyo, Japan) using Attractene Transfection Reagent (Qiagen, Tokyo, Japan). Cells were selected with hygromycin B (Invitrogen) and the surviving colonies were harvested and cultured independently. Those colonies were screened using a Luciferase Assay System (Promega KK) and a clone presenting the highest luciferase activity (JAR-Luc) was used in in vivo metastatic assays.

### Matrigel invasion assay

Cell invasion assays were performed in a BD Biocoat Growth Factor Reduced Matrigel Invasion Chamber (8 μm pre size; BD Biosciences, Tokyo, Japan) according to the manufacturer's protocol. Briefly, JAR or BeWo cells (5 × 10^4^ cells) were plated in 200 μL in the serum-free DMEM/F12 medium with or without different doses of the soluble ectodomain of TrkB (R&D Systems, Minneapolis, MN), a pan-specific Trk receptor inhibitor, K252a (Calbiochem, La Jolla, CA) [21], or the inactive plasma membrane nonpermeable K252b (Calbiochem) [22] to the upper chamber of 24-well transwell plate. Some cells were treated with both TrkB ectodomain and BDNF (R&D Systems). The lower chamber was filled with 500 μL DMEM/F12 with 10% FBS. Cells were allowed to invade the matrigel for 48 h in a humidified tissue culture incubator at 37°C and 5% CO_2_ atmosphere. The noninvading cells on the upper surface of the membrane were removed by scrubbing with cotton swabs, and the invaded cells on the lower surface of the membrane were stained by Diff-Quick stain (Sysmex Corp., Kobe, Japan). After staining, the invasion was determined by counting the number of stained cells on the membranes in 10 randomly selected, nonoverlapping fields at 200× magnification using a light microscope. To confirm that the changes were not due to differences in cell proliferation after treatment, cells were cultured in 96-well plate under the same treatments before determination of cell proliferation using a trypan blue exclusion test (Invitrogen). The number of viable cells was determined in a hemocytometer based on dye exclusion by viable cells.

### Matrix metallopeptidase activity assay, quantitative real-time RT-PCR, and Western blotting

To measure matrix metallopeptidase (MMP-2) transcript and protein levels, JAR cells were treated with or without the soluble ectodomain of TrkB (0.75 μg/mL), K252a (20 nmol/L), or K252b (20 nmol/L) in the 24-well transwell plate as described above. After 48 h of culture, conditioned medium was collected from the upper chamber, and then both invading and noninvading cells were stripped from the membrane and used for quantitative real-time reverse transcription polymerase chain reaction (RT-PCR). Quantitative real-time RT-PCR was performed using a SmartCycler (Takara, Tokyo, Japan) with primers and hybridization probes for β-actin as described [23]. Primers and probes of MMP-2 were as followed: sense 5′-CAACAGCTGCACTGATACCG-3′, antisense 5′-CCGTACTTGCCATCCTTCTC-3′, and probe 5′-6-carboxy-fluorescein-TCTGGTGCTCCACCACCTAC-AACTT-6-carboxy-tetramethyl-rhodamine-3′. Data were normalized based on β-actin transcript levels. After centrifugation of the conditioned medium at 8,000*g* for 5 min at 4°C, quantification of total and activated MMP-2 in the supernatant was performed using the MMP-2 Biotrack activity assay system (GE Healthcare, Little Chalfont, UK) according to the manufacturer's protocol. Briefly, standards and samples were incubated in microplate wells precoated with anti-MMP-2 antibody. After washing, active or total (proactive and active) levels of MMP-2 in samples were detected. In order to measure the total MMP-2 content, any bound MMP-2 in its proform was activated using *p*-aminophenylmercuric acetate (APMA). The standard is pro-MMP-2 which was activated in parallel for both types of sample. Active MMP-2 was detected without APMA treatment. The resultant color was read at 405 nm in a microplate spectrophotometer (Bio-Rad, Tokyo, Japan).

For Western blotting, conditioned media were concentrated using Amicon Ultra-0.5 mL Centrifugal Filter (Millipore, Billerica, MA). Samples were boiled for 5 min, and separated by electrophoresis under reducing conditions on 4–15% polyacrylamide gels with molecular weight markers (Precision Plus Protein Dual Color Standards; Bio-Rad) and transferred to polyvinylidene difluoride membranes. The membranes were incubated with anti-MMP2 polyclonal antibodies (Cell Signaling Technology, Danvers, MA) at 1:1000 dilution overnight at 4°C. Following incubation with horseradish peroxidase conjugated antirabbit IgG (GE Healthcare, Tokyo, Japan) at 1:10,000 dilution for 1 h at room temperature, MMP-2 was detected using SuperSignal West Femto (Pierce, Rockford, IL).

### In vivo invasion assay

To explore the roles of endogenous TrkB ligands during invasion of choriocarcinoma in vivo, the antitumor activity of K252a was determined in athymic female nude mice (BALB/c nu/nu) (CLEA Japan, Tokyo, Japan) bearing JAR tumors. The care and use of animals were approved by the Animal Research Committee, Akita University School of Medicine. JAR cells (0.1 mL, 5 × 10^6^ cells) were implanted subcutaneously on the right frank of female nude mice at 4–5 weeks of age as described [20]. Treatment was initiated when the tumor volume reached ∼60 mm^3^. Intraperitoneal administration of K252a dissolved in physiological saline (500 μg/kg) was performed every 3 days. For negative controls, treatment with K252b (500 μg/kg) or vehicle alone was used. The doses of K252a and K252b chosen for these experiments were based on previous studies [20, 24]. Tumor volumes were measured daily using the formula: tumor volume (mm^3^) = length × (width)^2^ × 0.5. Mice were killed after 9 days of treatment and subcutaneous tumors were also resected together with surrounding tissues to evaluate cellular invasion. Hematoxylin and eosin (H&E) staining was performed to visualize cell invasion from the tumors into surrounding tissues.

### In vivo metastasis assay

To establish a model for monitoring of the antitumor efficacy of K252a in choriocarcinoma metastasis, JAR-Luc cells (0.1 mL, 1.2 × 10^6^ cells) were injected intravenously into female severe combined immunodeficiency (SCID) mice (CLEA Japan) at 6–8 weeks of age and their metastasis monitored by using in vivo bioluminescence luciferase imaging (IVIS system; Xenogen, Alameda, CA). Treatment was initiated in animals at 2 weeks after cell injection when photon signals were enabled for detection in SCID mice. Mice bearing metastatic tumors were treated with an intraperitoneal injection of vehicle, K252a (500 μg/kg), or K252b (500 μg/kg) every 3 days as described above. These animals were imaged before and at the end of treatment. Some animals were treated at the same time of cell injection and the Trk inhibitors were administrated intraperitoneally every 3 days. At 2 weeks after cell injection, the presence of tumor metastasis tumor was detected using the bioluminescent imaging.

For bioluminescent imaging, mice were anesthetized with isoflurane (Dainippon Pharmaceutical Co., Ltd., Tokyo, Japan) and then were intraperitoneally injected with firefly terminal deoxynucleotidyl transferase mediated dUTP nick end labeling (TUNEL) d-luciferin (Promega KK) dissolved in sterile phosphate buffered saline (PBS) (150 mg/kg). Images were obtained at 25 min after injection followed by 1 min exposure in the imaging system. Bioluminescence from the region of interest (ROI) was defined over the contour of individual tumors to include all photon emission from the entire tumor, and the data of photon signals were expressed as fold increases relative to controls. Images in the same figure were formatted with the identical rainbow color–coded scale for visual assessment.

Mice were killed after 9 days of treatment, and blood samples were collected from all tested mice to determine serum hCG-β levels using RIA conducted at Mitsubishi BCL (Tokyo, Japan), as described [20]. The minimum detectable level of the assay is 0.1 ng/mL with intra- and interassay coefficients of variations of 4.5% and 6.3%, respectively. Metastatic lung tumors were also excised from animals. In addition to H&E staining, in vivo cell proliferation and apoptosis were evaluated by immunostaining of proliferating cell nuclear antigen (PCNA) and the TUNEL assay, respectively.

For PCNA immunostaining, tumor samples were fixed in Bouin's solution for 2 h. After deparaffinization and dehydration, antigen retrieval was performed in samples by autoclave heating at 121°C for 10 min in 10 mmol/L citrate buffer (pH 6) for 3 min. Endogenous peroxidase activities were quenched with 0.3% hydrogen peroxidase in methanol for 30 min. After blocking with 10% BSA Tris-buffered saline (Sigma, St. Louis, MO) for 30 min, slides were incubated with mouse anti-PCNA monoclonal antibodies (Cell Signaling Technology) at 1:4000 dilution overnight at 4°C. After three washes in Tris-buffered saline, slides were incubated with biotinylated antimouse secondary antibodies (Invitrogen) for 30 min at room temperature. After three washes, bound antibodies were visualized using a Histostain SP kit (Invitrogen). For negative controls, the primary antibody was replaced by nonimmune mouse IgG1 (Dako, Carpinteria, CA).

For the TUNEL assay, tumor samples were fixed in 4% paraformaldehyde (Sigma) for 1 h at room temperature. After deparaffinization and dehydration, slides were treated with proteinase K (Roche Applied Science, Indianapolis, IN) for 30 min at room temperature, and then incubated with TUNEL reagent (Roche) for 1 h at 37°C in the dark. For positive controls, slides were treated with 50 μg/mL deoxyribonuclease I (Invitrogen) for 20 min at 37°C prior to the TUNEL reaction. For negative controls, slides were incubated with TUNEL reagents in the absence of the enzyme terminal deoxynucleotidyl transferease. Counterstaining was performed by incubating placentas with 100 μg/mL propidium iodide (Sigma) and 50 μg/mL RNase A (Ambion, Inc., Austin, TX) for 20 min at room temperature. After washing, slides were mounted with Slowfade light antifade solution (Invitrogen). The fluorescence signals in samples were visualized using an epifluorescent microscope (Olympus Corp., Tokyo, Japan).

Caspase-3/7 activities in excised tumor samples were measured by the Caspase-Glo 3/7 assay, as described [20]. Data were normalized by protein concentrations and represented as fold increases relative to controls. To evaluate the effects of Trk receptor inhibitors on angiogenesis in metastatic tumors, CD31 (cluster of differentiation 31) and VEGF-A (vascular endothelial growth factor A) transcript levels were measured using quantitative real-time RT-PCR as described earlier. Validated Taqman gene expression assay was used to quantify the expressions of CD31 and VEGF-A (Applied Biosystems, Forster City, CA). Data were normalized based on β-actin transcript levels.

### Statistical analysis

Experiments were repeated at least three times. Differences were evaluated using one-way analysis of variance (ANOVA), followed by Fisher's protected least significant difference test. Data are mean ± SEM.

## Results

### In vitro inhibition of endogenous TrkB signaling suppressed choriocarcinoma cell invasion

**Figure 1:**
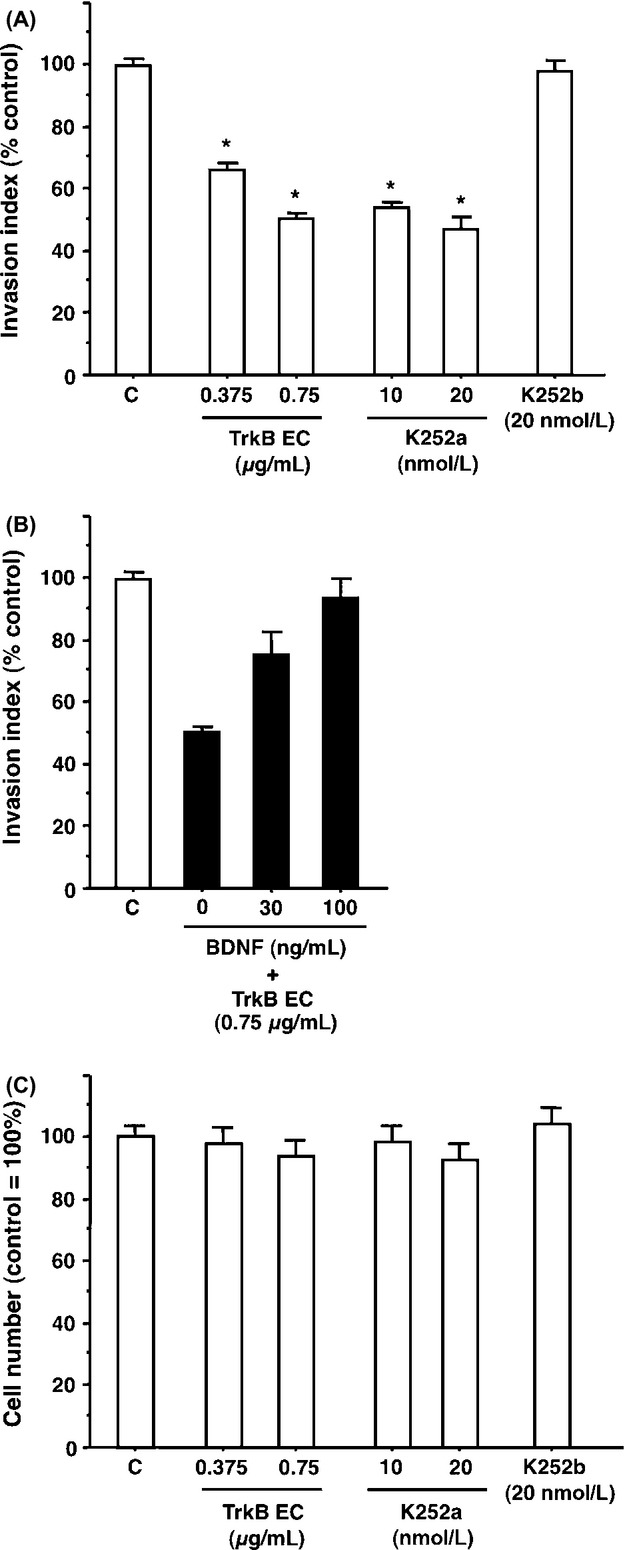
Effects of inhibition of endogenous TrkB signaling on JAR cell invasion. (A and B) Suppression of cell invasion after inhibition of endogenous TrkB signaling (Mean ± SEM, *n* = 6–12). Cells were cultured in medium alone (control, C), with different doses of TrkB ectodomain (TrkB EC), K252a, or with the inactive plasma membrane nonpermeable K252b (20 nmol/L) on matrigel-coated transwell plate for 48 h. Some cells were cotreated with TrkB EC (0.75 μg/mL) and brain-derived neurotrophic factor (BDNF). The invasive cells on the lower surface of the membrane were counted under a microscope. Results are expressed based on invasion index calculated as percentages relative to controls. *P < 0.05 versus control group. (C) Cell proliferation after inhibition of endogenous TrkB signaling. Cells were seeded into 96-well culture plates and treated without (control, C) or with TrkB, K252a, or K252b in the cell invasion assay. At 48 h after culture, cell numbers were determined using the Trypan blue exclusion test (Mean ± SEM, *n* = 6–12). All experiments were repeated at least three times.

**Figure 2:**
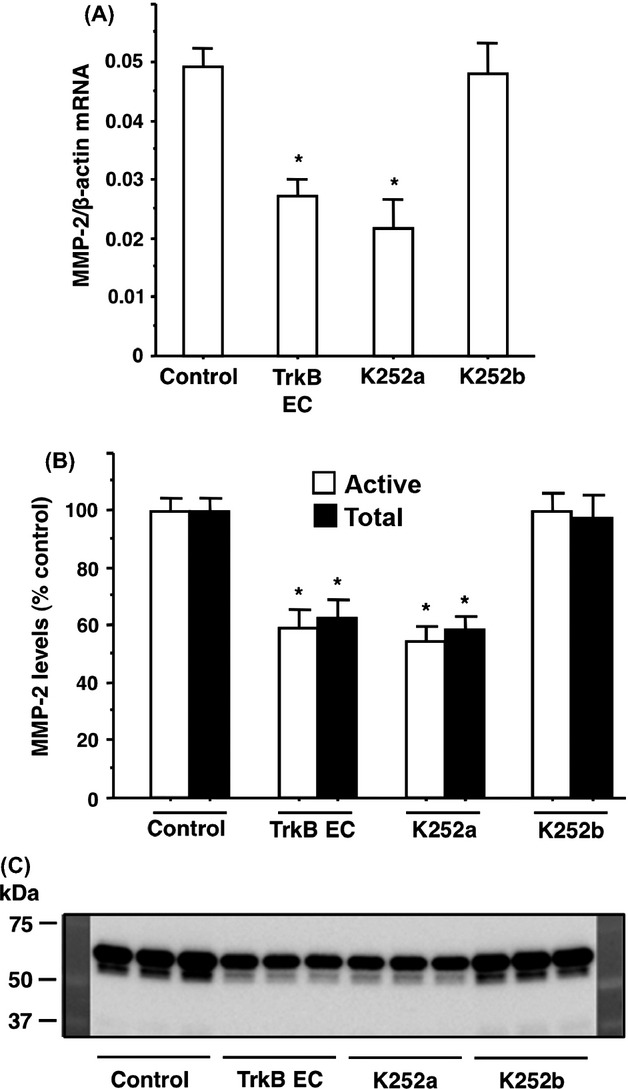
Suppression of MMP-2 expression in JAR cell after inhibition of endogenous TrkB signaling. Cells were cultured in medium alone (control), with TrkB ectodomain (TrkB EC, 0.75 μg/mL), K252a (20 nmol/L), or K252b (20 nmol/L) on matrigel-coated transwell plate for 48 h to evaluate the effects of inhibition of endogenous TrkB signaling on MMP-2 mRNA expression (A) and protein (B and C) expression. MMP-2 transcript levels in cells were quantified using real-time RT-PCR and normalized using those for β-actin in the same sample. Conditioned medium was also collected from the upper chamber and subjected to measurement of total and activated MMP-2 proteins by ELISA based on the MMP-2 activity assay (mean ± SEM, *n* = 4) and Western blotting (*n* = 3). Results are expressed as percent of controls. *P < 0.05. All experiments were repeated at least three times.

We previously showed the expression of both TrkB ligands and receptors in JAR cells, a choriocarcinoma cell line [20]. To investigate the effects of endogenous TrkB signaling on choriocarcinoma invasion, we determined changes in cellular potential for invasion in cultured JAR cells after treatment with TrkB ectodomain and K252a using the Matrigel invasion assay. In controls, invaded cells on the lower surface of the membrane were evident at 24 h and reached maximum levels at 48 h of cultures (data not shown). Treatment with the TrkB ectodomain or K252a, but not the inactive K252b, decreased the number of invaded cells (Fig. 1A). Because soluble TrkB ectodomain suppresses TrkB signaling by binding BDNF derived from cultured cells, its inhibitory effect on cell invasion was reversed by excess BDNF added exogenously (Fig. 1B). Although JAR cell proliferation was suppressed by treatment with TrkB ectodomain and K252a [20], the tested doses did not affect cell proliferation (Fig. 1C), indicating that observed changes in cell invasion were not due to differences in cell proliferation. Using BeWo cells, a second choriocarcinoma cell line, we obtained similar results showing decreases in the number of invaded cells following treatment with the TrkB ectodomain or K252a, but not with the inactive K252b (Fig. S2A). In these tests, cell proliferation was not affected (Fig. S2C). Furthermore, the inhibitory effect of TrkB ectodomain on cell invasion was reversed following treatment with exogenous BDNF (Fig. S2B). To further characterize inhibition of cell invasion following the suppression of endogenous TrkB signaling in choriocarcinoma cells, the levels of a cell invasion marker, MMP-2, were measured in JAR cells. As shown in Figure 2A, treatment with either the TrkB ectodomain or K252a, but not with the inactive K252b, decreased MMP-2 transcript levels in cultured JAR cells (Fig. 2A). Furthermore, enzyme-linked immunosorbent assay (ELISA)-based assay detected decreases in the levels of both total and active MMP-2 proteins in the conditioned medium of cell cultures following treatment with different inhibitors (Fig. 2B). Observed changes in protein levels of full-length proenzyme (Fig. 2C, upper band) and cleaved active enzyme (Fig. 2C, lower band) were also confirmed by Western blotting analyses.

### In vivo effects of a Trk receptor inhibitor on choriocarcinoma cell invasion

**Figure 3:**
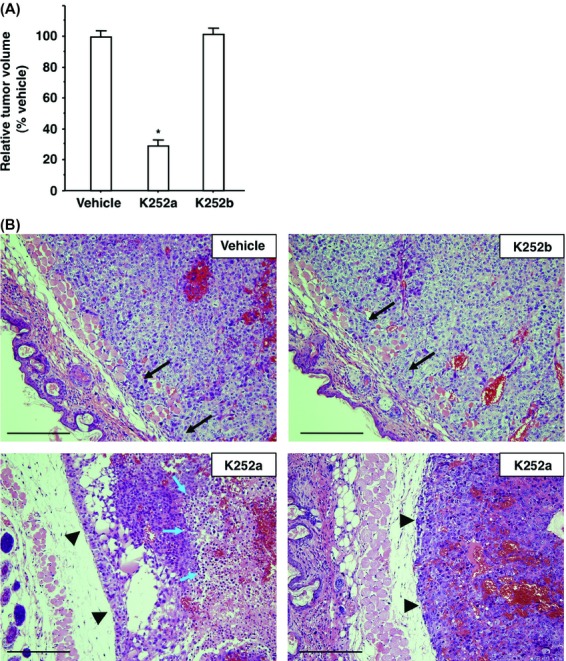
Suppression of in vivo tumor invasion of JAR xenografts by the Trk receptor inhibitor. Nude mice bearing tumors were treated without (vehicle) or with K252a or K252b (500 μg/kg) every 3 days. All experiments were repeated at least three times. (A) Tumor growth in mice at 9 days after the beginning of treatment. Volume of each tumor at the first day of grating when mice received treatment was set as a relative tumor volume of 1. (mean ± SEM, *n* = 10–12) *P < 0.05. (B) Histology of JAR cell invasion into the borderline area between tumors and host tissues. Tissue samples were obtained from animals at 9 days after the beginning of treatment. Represented images were obtained from resected tumors. Hematoxylin and eosin (H&E) staining shows invasion of tumor cells into the muscle layer (black arrows) in vehicle and K252b-treated groups, whereas clear boundary between tumor cells and muscle layer (arrow heads) is evident after K252a treatment. In K252a-treated animals, H&E staining also shows decreases in the number of mitotic cells and increases in number of cells with chromatin condensation (blue arrows). Scale bars, 200 μm.

To determine whether the observed inhibition of choriocarcinoma cell invasion in vitro by TrkB inhibitors could be extended into antitumor activity in vivo, K252a was administrated to athymic nude mice with tumor xenografts of JAR cells. When the tumor volume reached ∼60 mm^3^, animals were given K252a or K252b at 500 μg/kg every 3 days. At 9 days after treatment, local invasion at the original grafted tumor site was evaluated histologically in dissected subcutaneous tumors together with surrounding tissues. Consistent with a previous study [20], tumor growth was suppressed following treatment with K252a, but not K252b, due to the inhibitory effect of K252a on cell proliferation (Fig. 3A). Although we could not find any apparent tumor metastasis in all tested animals similar to earlier studies [20, 25], cells were invaded vertically into the subcutaneous muscle layer, resulting in an obscure boundary between tumor and muscle layer in controls (Fig. 3B, vehicle, black arrows). In contrast, K252a-treated animals showed no apparent vertical invasion into the muscle layer (Fig. 3B, K252a, arrowheads), suggesting inhibition of cell invasion following the suppression of endogenous TrkB signaling. Furthermore, histopathological examination revealed decreases in mitotic activity and increases in number of cells with chromatin condensation within tumors of K252a-treated mice (Fig. 3B, blue arrows), suggesting suppression of cell proliferation and survival. Consistent with previous studies [8, 20], no side effect was observed throughout experiments in all tested animals, and no change in body weight was found in K252a-treated group during studies (vehicle [*n* = 10], 21.45 ± 0.68 g; K252a [*n* = 12], 20.56 ± 0.52 g; and K252b [*n* = 10], 20.72 ± 0.55 g). Of importance, treatment with the inactive plasma membrane nonpermeable K252b was ineffective for all parameters tested.

### In vivo effects of a Trk receptor inhibitor on choriocarcinoma cell metastasis

To assess the potential of suppressing endogenous TrkB signaling for a treatment for choriocarcinoma metastasis, JAR-Luc cells expressing firefly luciferase were administrated intravenously into SCID mice as an in vivo model of metastasis [26]. Under in vivo bioluminescence luciferase imaging, photon signals were negligible at 1 week after injection of the cells (data not shown), but were detectable after 2 weeks in lung, liver, and/or ovary of 80% (16/20) of animals (Fig. S1). Thus, K252a treatment was initiated in animals with detectable photon signals at 2 weeks after cell injection to determine the effects of suppressing endogenous TrkB signaling on metastatic tumor growth. Also, some animals were treated with K252a beginning at the same time of cell injection for 2 weeks to evaluate their effectiveness to suppress metastasis to distant organs. In mice treated with different drugs from the same time of cell injection, photon signals were detected in 73% (11/15) and 80% (12/15) of animals treated with vehicle and K252b, respectively. In contrast, only 7% of animals (1/15) treated with K252a were positive for photon signals.

**Figure 4:**
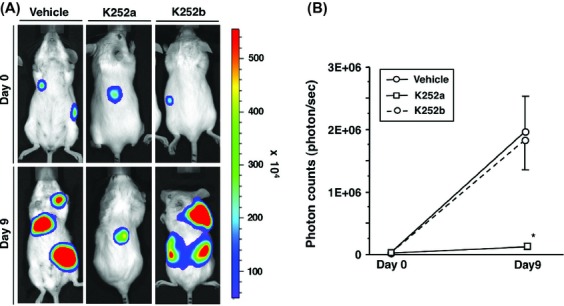
Suppression of in vivo tumor metastasis by the Trk receptor inhibitor in severe combined immunodeficiency (SCID) mice. JAR cells transfected with the firefly luciferase vector (JAR-Luc cells) were injected intravenously. At 2 weeks after cell injection, metastasis was detected by using in vivo bioluminescence luciferase imaging. Animals bearing metastatic tumors were treated without (vehicle) or with K252a or K252b (500 μg/kg) every 3 days. (A and B) Metastatic tumor growth in mice treated with K252a. Represented bioluminescent images were obtained at 9 days after treatment. Photon counts were measured before (Day 0) and at the end of treatment (Day 9) (mean ± SEM, *n* = 10). *P < 0.05 versus vehicle group. Experiments were repeated three times with comparable results.

Because there were multiple metastatic lesions found in animals, photon signals from separated lesions were integrated for the measurement of photon counts in each animal. Because mice with metastasis began to die after 12 days of treatment, the effects of K252a treatment on the growth of metastatic tumor were evaluated by measuring bioluminescence in the mice at 9 days after treatment (*n* = 10 in each group). In contrast to increased areas of detectable luminescence in vehicle- and K252b-treated animals, the luminescence-positive areas were limited in mice with K252a treatment (Fig. 4A). Furthermore, K252a administrations decreased the levels of photon signals, whereas K252b was ineffective (Fig. 4B). No side effect was observed throughout experiments in all tested animals.

**Figure 5:**
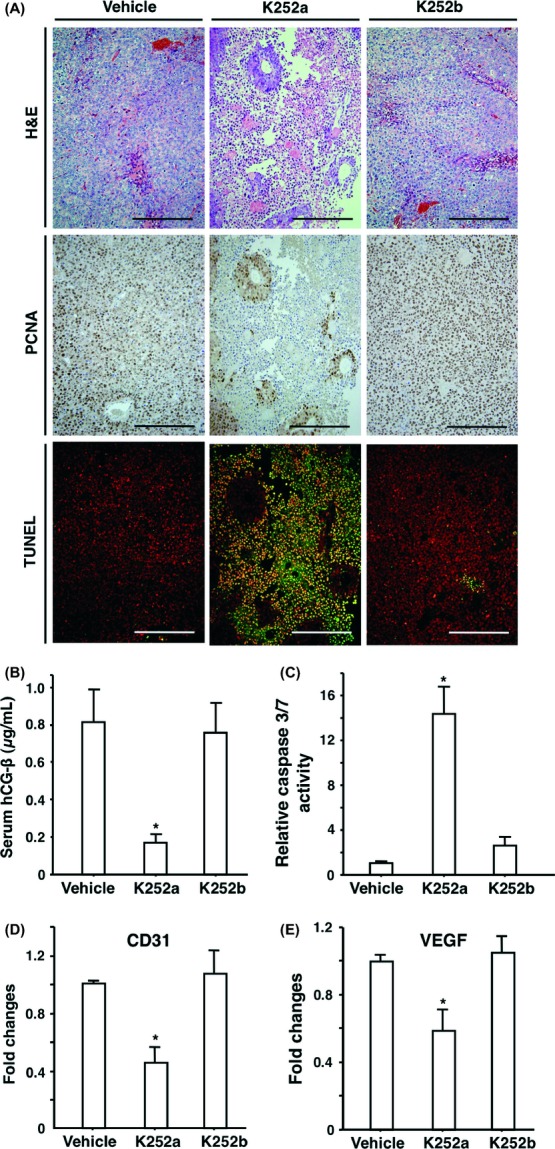
Suppression of in vivo tumor proliferation, survival, and angiogenesis of metastatic tumors by the Trk receptor inhibitor in severe combined immunodeficiency (SCID) mice. (A) Histological characterization of cell proliferation and apoptosis in tumors. Detection of cell proliferation and DNA fragmentation was performed using proliferating cell nuclear antigen (PCNA) immunostaining and in situ TUNEL staining, respectively. Animals bearing metastatic tumors were treated without (vehicle) or with K252a or K252b (500 μg/kg) every 3 days. Represented images were obtained from resected lung tumors at 9 days of treatment. Hematoxylin and eosin (H&E) staining shows decreases in number of mitotic cells and increases in number of cells with chromatin condensation after K252a treatment. PCNA signals (brown) were decreased, whereas TUNEL stained nuclei (green fluorescence) were increased following K252a treatment. Scale bars, 200 μm. (B–E) Serum human chorionic gonadotropin (hCG)-β levels (B), and caspase-3/7 activities (C) as well as transcript levels for CD31 (D) and vascular endothelial growth factor A (VEGF-A) (E) in metastatic lung tumors of the mice. Serum and tissue samples were obtained from the mice at 9 days of treatment. Data were normalized based on protein concentrations for caspase-3/7 activities, whereas CD31 and VEGF-A transcript levels were normalized using β-actin mRNA levels in the same samples, and expressed as fold increases relative to controls (vehicle alone) and normalized to 1 (mean ± SEM, *n* = 10). *P < 0.05 versus control group. All experiments were repeated at least three times.

Excised metastatic tumors were further examined to evaluate in vivo effects of K252a. After K252a treatment, histopathological examination revealed decreases in cellular mitotic activity and increases in number of cells with chromatin condensation within tumors of K252a-treated mice (Fig. 5A, upper panel), suggesting suppression of cell proliferation and survival. Reduction in PCNA staining after K252a treatment confirmed its effects on the suppression of cell proliferation (Fig. 5A, middle panel). Serum hCG-β levels were decreased by 80% in metastatic tumors after K252a treatment (Fig. 5B), indicating a loss of cellular viability to synthesize this tumor marker. Also, induction of apoptosis in tumor tissues was confirmed by the TUNEL assay (Fig. 5A, lower panel). Quantitative analyses of apoptosis showed a 14.5-fold increase in caspase-3/7 activity within the tumors of K252a-treated mice (Fig. 5C). Furthermore, K252a treatment decreased transcript levels of an endothelial cell marker, CD31, and a proangiogenic factor, VEGF-A (Fig. 5D) in metastatic tumors, suggesting inhibition of angiogenesis. In these experiments, treatment with the inactive plasma membrane nonpermeable K252b was ineffective for all parameters tested.

## Discussion

Following in vitro and in vivo studies of choriocarcinoma cells, we demonstrated important roles of TrkB signaling in cell invasion and metastasis. Suppression of endogenous TrkB signaling by Trk inhibitors not only led to inhibited choriocarcinoma cell invasion but also decreased their metastasis to distant organs, accompanied by suppressed growth of metastatic tumors. Our previous study demonstrated that suppression of endogenous TrkB signaling also inhibited cell proliferation and survival in choriocarcinoma cells [20]. These findings indicated that TrkB signaling is a valuable antitumor target with a therapeutic potential for both early and advanced stages of choriocarcinoma. Because both TrkB receptor and its ligands, BDNF and NT-4, were expressed in JAR cells [20], TrkB ligands could act on the TrKB receptors expressed in JAR cells via an autocrine mechanism. Using different doses of Trk inhibitors not affecting cell proliferation, we demonstrated the autocrine roles of TrkB signaling in choriocarcinoma cell invasion. Because treatment of JAR cells with TrkB siRNA inhibited cell proliferation [20], it is difficult to evaluate the roles of TrkB signaling in cell invasion and MMP-2 expression using TrkB siRNAs. Similar to the siRNA study, inhibition of TrkB signaling at high doses of TrkB ectodomain or K252a suppressed both cell proliferation and invasion simultaneously, leading to an inappropriate interpretation of results. In contrast, we could demonstrate suppression of cell invasion without affecting cell proliferation using lower doses of these inhibitors.

Using the present in vivo model of choriocarcinoma, we further demonstrated that suppression of TrkB signaling by the Trk receptor inhibitor, K252a, inhibited cell invasion of JAR xenografts in nude mice without obvious side effects in host animals.

Among members of the MMP family, MMP-2 and MMP-9 are expressed in trophoblast cells and act as key enzymes in their invasion [27]. In JAR cells, MMP-2 is the predominant form with a much lower expression of MMP-9 [28]. We measured MMP-2 levels and found decreased MMP-2 expression in cultured JAR cells following suppression of endogenous TrkB signaling. Using the TrkB ectodomain and the Trk receptor inhibitor, K252a, we showed the ability of endogenous TrkB ligands to promote the invasion of the choriocarcinoma cells. Although K252a is a pan-specific Trk receptor inhibitor and could have potential actions other than the suppression of TrkB signaling, the observed inhibitory effect is likely specific for the TrkB receptor because the other paralogous receptor, TrkC, was not detectable in the choriocarcinoma cells [20] and the highly specific, soluble ectodomains of TrkB exhibited similar inhibitory effects as K252a on cell invasion.

Choriocarcinoma is highly invasive in patients and several lines of evidence indicated that growth factors regulate choriocarcinoma cell invasion. hCG is an important tumorigenic factor and shown to stimulate the invasion of JEG-3 choriocarcinoma cells accompanied by increases in MMP-2 and MMP-9 levels [29]. However, our previous data demonstrated that decreases in production of hCG following hCG antisense transfection in JAR cells did not affect BDNF levels [20], suggesting that BDNF/TrkB signaling acts on choriocarcinoma cell invasion independent of hCG. Other factors, such as signaling mediated by Ephrin-Eph A [30], TGF-β1 [31], and interleukin-11 [32], also promoted the invasion of JEG-3 cells, whereas interleukin-12 inhibits JEG-3 cell invasion through regulating the expression of MMP-9 and metalloproteinases-1 [33]. Among these growth factor signaling pathways, BDNF/TrkB signaling showed promise for the development of novel anticancer therapies due to its potential to inhibit both cell invasion and proliferation.

Choriocarcinoma is prone to metastasize hematogenously to multiple organs, mainly lung, liver, and brain. Because no clear signs of tumor metastasis were observed in our tumor xenograft model of athymic nude mice [20], we hypothesized that a more sensitive model is needed. We injected JAR-Luc cells expressing firefly luciferase intravenously into SCID mice to establish a more sensitive in vivo model of choriocarcinoma metastasis. In this model, metastasis was detected in lung, liver, and/or ovary of 80% of control animals at 2 weeks after tumor cell administration, although brain metastasis was not found. Of importance, K252a treatment suppressed tumor metastasis to distant organs by >70%, suggesting its therapeutic potential. Although the inhibition of metastasis was likely caused by suppression of endogenous TrkB signaling during tumorigenesis, exact cellular mechanisms remain to be determined. Using the noninvasive bioluminescence assay, administration of K252a was found to be effective in suppressing the growth of metastatic tumor by decreasing in cell proliferation, viability, and survival.

Due to the importance of angiogenesis in tumor growth, antiangiogenic therapy is a promising therapeutic option for cancer patients [34]. Accumulating evidences indicate that BDNF may act as an angiogenic factor in normal [17, 35, 36] and tumor tissues [37]. In human hepatocellular carcinoma, BDNF stimulated tumor growth via promoting angiogenic functions of endothelial cells expressing TrkB [37]. Because it is difficult to quantitatively evaluate angiogenesis based on histology, levels of endothelial cell marker, CD31, and proangiogenic factor, VEGF-A, were measured. We found that K252a treatment decreased transcript levels for CD31 in metastatic tumors. Consistent with BDNF/TrkB signaling-mediated induction of VEGF-A activity in neuroblastoma cells [38], levels of VEGF-A mRNA were also decreased in metastatic tumors by the suppression of endogenous TrkB signaling. These results suggested the contribution of BDNF/TrkB signaling in angiogenesis of metastatic choriocarcinoma.

Several lines of evidence indicated the stimulatory effects of TrkB signaling on the metastasis of endometrial [39, 40] and ovarian [11, 41–44] cancers. However, the roles of TrkB signaling in cell proliferation and survival at primary tumor site have not been elucidated. Here, we have demonstrated roles of the BDNF/TrkB signaling system in choriocarcinoma cell invasion and metastasis, in addition to its roles in tumor growth at primary site [20]. TrkB signaling is also known to contribute to resistance to anoikis (detachment-mediated apoptosis) [18, 19], which promotes cancer cell survival in circulation and dispersion to distant organs. Collectively, inhibition of this signaling system could provide the basis to develop novel therapies for choriocarcinoma from early to advanced stages to complement currently available chemotherapies.

## Supporting Information

**Figure S1.** Macroscopic and microscopic images of metastatic tumors after injection of JAR-Luc cells into SCID mice. At 23 days after intravenous injection of the cells, animals were dissected to find metastatic tumors (*n* = 20). Experiments were conducted five times. (A) Macroscopic appearance of tumor metastasis in SCID mice. Metastatic tumors were detected in lung (yellow arrows), ovary (black arrow), and liver (arrow heads). (B and C) Microscopic images of metastatic tumors in lung. Lower magnification of H&E staining (B) shows normal pulmonary alveoli in left side and a round solid tumor with rich blood vessels in right side. Scale bar, 500 μm. High-magnification image (C) indicates venous invasion (arrow heads) and boundary of the tumor (arrows). Scale bar, 200 μm.

**Figure S2**. Effects of inhibition of endogenous TrkB signaling on BeWo cell invasion. (A and B) Suppression of cell invasion after inhibition of endogenous TrkB signaling (Mean ± SEM, *n* = 6–12). Cells were cultured in medium alone (control, C), with different doses of TrkB ectodomain (TrkB EC), K252a, or with the inactive plasma membrane nonpermeable K252b (20 nmol/L) for 48 h. Some cells were cotreated with TrkB EC (0.75 μg/mL) and BDNF. The invasive cells on the lower surface of the membrane were counted under a microscope. Results are expressed based on invasion index calculated as percentages relative to controls. **P* < 0.05 versus control group. (C) Cell proliferation after inhibition of endogenous TrkB signaling. Cells were seeded into 96-well culture plates and treated without (control, C) or with TrkB, K252a, or K252b in the cell invasion assay. At 48 h after culture, cell numbers were determined using the Trypan blue exclusion test (Mean ± SEM, *n* = 12). All experiments were repeated at least three times.
