# Supplementary figures and images for: Suppression of choriocarcinoma invasion and metastasis following blockade of BDNF/TrkB signaling
Source: Cancer Med. 2013 Nov 7;2(6):849–61. doi: 10.1002/cam4.158 (PMC3892389; doi:10.1002/cam4.158)

## Slide 1
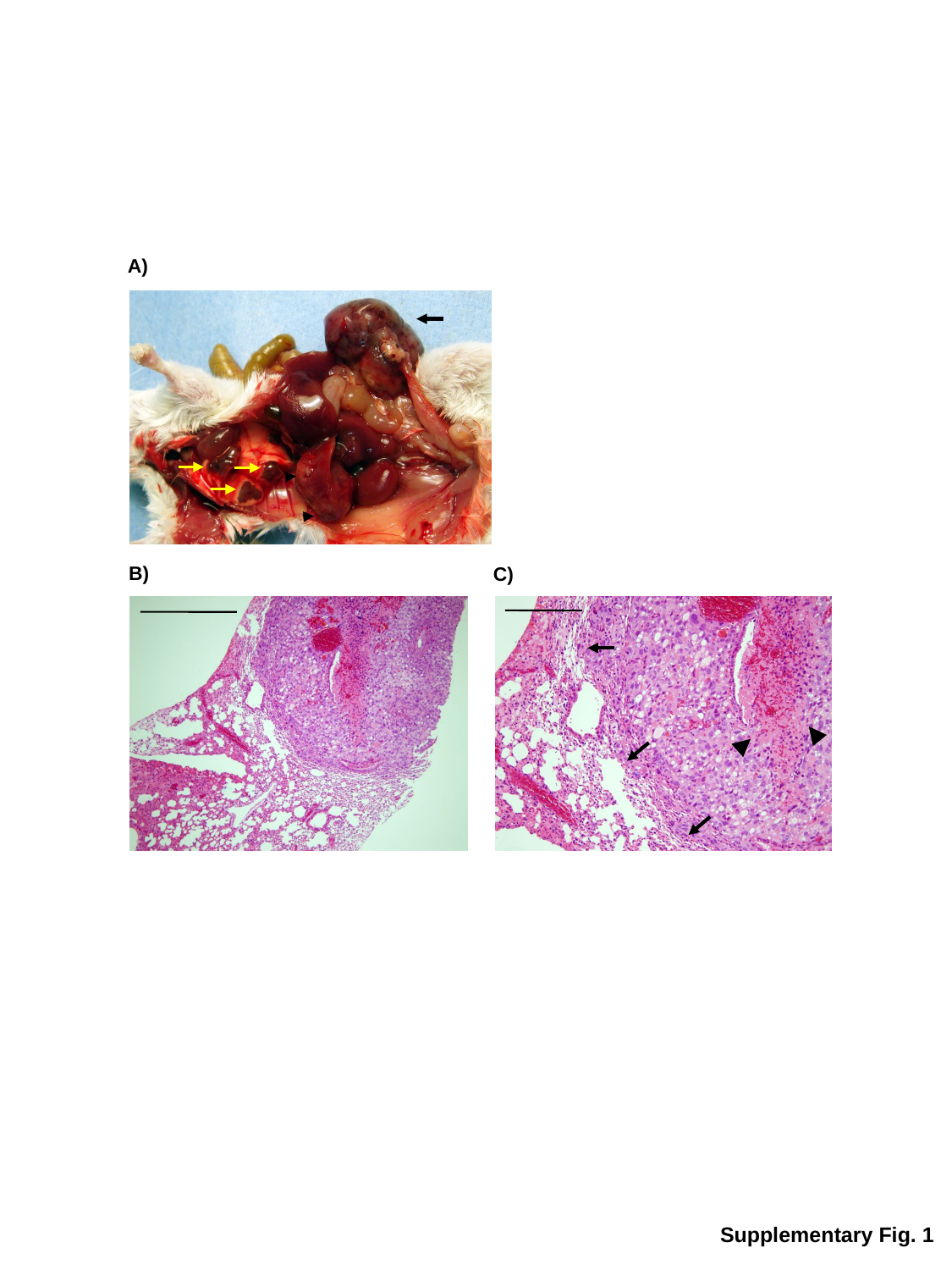

A)
B)
C)
Supplementary Fig. 1

Supplement: Supplementary file 1 [file cam40002-0849-SD1.pptx]

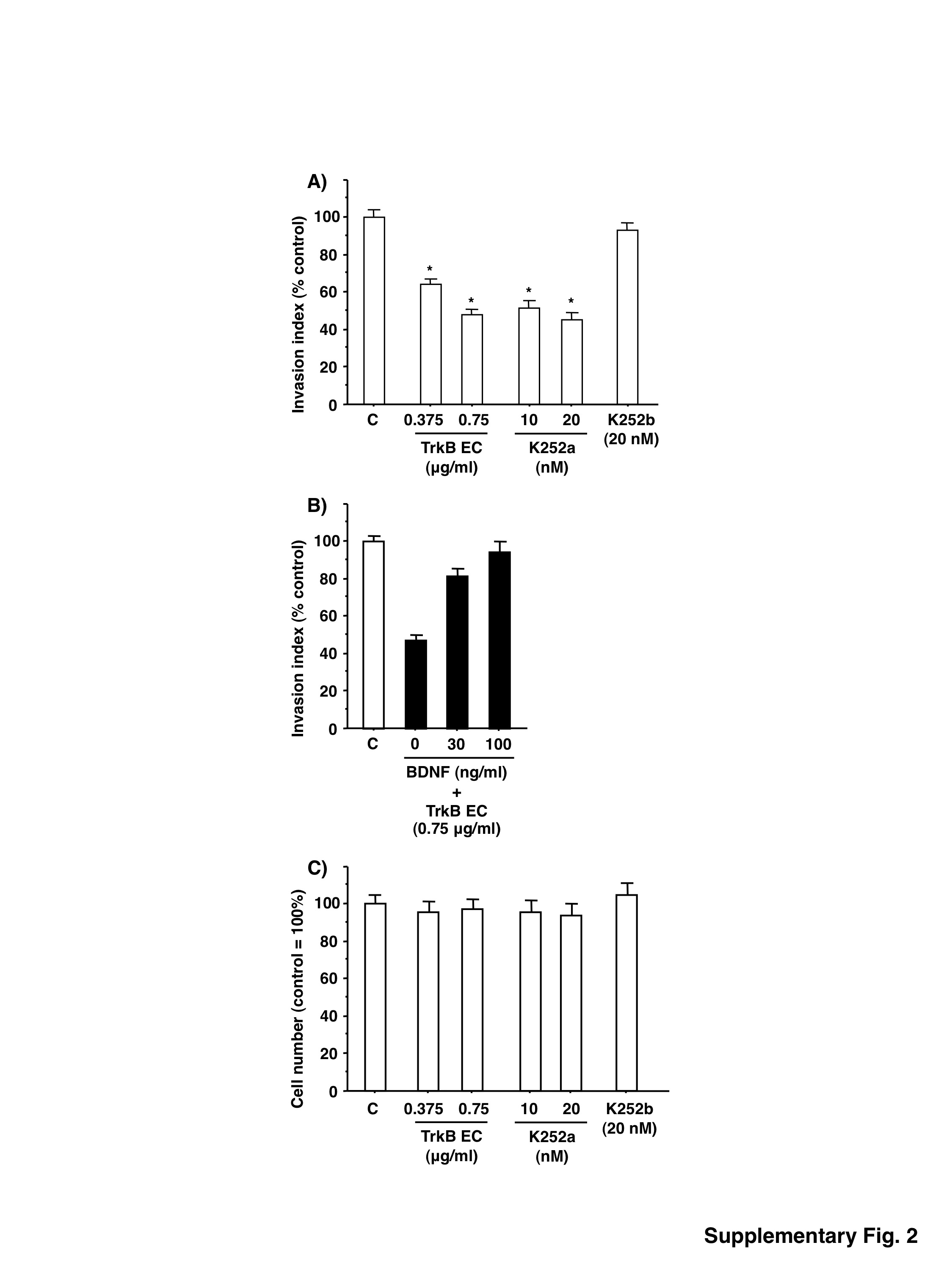

Supplement: Supplementary file 2 [file cam40002-0849-SD2.jpg]
